# First Steps towards a near Real-Time Modelling System of *Vibrio vulnificus* in the Baltic Sea

**DOI:** 10.3390/ijerph20085543

**Published:** 2023-04-17

**Authors:** Eike M. Schütt, Marie A. J. Hundsdörfer, Avril J. E. von Hoyningen-Huene, Xaver Lange, Agnes Koschmider, Natascha Oppelt

**Affiliations:** 1 Earth Observation and Modelling, Department of Geography, Kiel University, 24118 Kiel, Germany; 2 Molecular Microbiology, Institute for General Microbiology, Kiel University, 24118 Kiel, Germany; 3 Leibniz Institute for Baltic Sea Research Warnemünde, 18119 Rostock, Germany; 4 Business Informatics and Process Analytics, University of Bayreuth, 95447 Bayreuth, Germany

**Keywords:** *Vibrio vulnificus*, Baltic Sea, near real-time modelling, St. Nicolas House Analysis, network inference, public health risk, climate change

## Abstract

Over the last two decades, *Vibrio vulnificus* infections have emerged as an increasingly serious public health threat along the German Baltic coast. To manage related risks, near real-time (NRT) modelling of *V. vulnificus* quantities has often been proposed. Such models require spatially explicit input data, for example, from remote sensing or numerical model products. We tested if data from a hydrodynamic, a meteorological, and a biogeochemical model are suitable as input for an NRT model system by coupling it with field samples and assessing the models’ ability to capture known ecological parameters of *V. vulnificus*. We also identify the most important predictors for *V. vulnificus* in the Baltic Sea by leveraging the St. Nicolas House Analysis. Using a 27-year time series of sea surface temperature, we have investigated trends of *V. vulnificus* season length, which pinpoint hotspots mainly in the east of our study region. Our results underline the importance of water temperature and salinity on *V. vulnificus* abundance but also highlight the potential of air temperature, oxygen, and precipitation to serve as predictors in a statistical model, albeit their relationship with *V. vulnificus* may not be causal. The evaluated models cannot be used in an NRT model system due to data availability constraints, but promising alternatives are presented. The results provide a valuable basis for a future NRT model for *V. vulnificus* in the Baltic Sea.

## 1. Introduction

*Vibrio* spp. are ubiquitous members of the ocean and freshwater microbial communities. More than 100 different *Vibrio* species have been identified [1]. They occur free-living in the water column or are attached to biotic or abiotic surfaces [2,3]. *Vibrio* spp. play an important role in marine nutrient cycles [4,5], and some species are known to form symbiotic relationships with marine animals (e.g., *Aliivibrio fischeri* and squids) [6]. About a dozen *Vibrio* spp. are human pathogens [1,7,8]. For example, two serogroups of *Vibrio cholerae* (O1 and O139) are the causative agents of the intestinal disease cholera [1]. Non-O1, non-O139 *V. cholera* strains and other *Vibrio* spp. cause vibriosis, with symptoms ranging from gastroenteritis to skin and soft tissue infections with necrotizing fasciitis, septicaemia, or even fatal septic multi-organ failure [7,9]. Infection pathways include handling and consumption of contaminated raw seafood as well as direct exposure to seawater [1,10].

Vibriosis outbreaks in Northern Europe are often attributed to *V. alginolyticus*, *V. cholerae* (*non-O1/non-O139*), *V. parahaemolyticus*, and *V. vulnificus* [11,12,13]. The number of incidents is generally low. However, infections with the species *V. vulnificus* are often severe (>90% of all cases) and have high mortality rates [9,12,13]. Therefore, vibriosis, particularly vibriosis linked to *V. vulnificus*, is a public health concern [13]. Empirical evidence of increased infection rates in warmer summers indicates that this problem will gain traction in the coming decades [11,12,13,14,15,16].

Understanding the ecology of *Vibrio*, which causes vibriosis, particularly of *V. vulnificus*, and predicting their abundance is an important component for the effective management of Vibrio-related public health risks [17]. With growth rates of up to several generations per hour, *Vibrio vulnificus* is known to form short but intense blooms under favorable conditions [18,19]. It has commonly been observed that favourable conditions consist of water temperatures above approximately 20 °C and salinity ranging from 5–25 practical salinity units (PSU) [19,20,21,22,23]. The predictive power of other variables, such as turbidity, chlorophyll a (Chl), dissolved organic carbon (DOC), or concentrations of nutrients, varies between study regions, indicating that limiting factors differ between habitats [2].

The Baltic Sea has low salinity and one of the highest warming rates in marine ecosystems worldwide [24]; thus, it is considered a high-risk environment for vibriosis infections [13,14]. In recent years, infection numbers have increased significantly along the German Baltic coast, particularly during heatwaves [12,15]. Infection risk management along the German Baltic Sea includes regular *V. vulnificus* quantification from water samples at seven beaches in the federal state of Mecklenburg–Western Pomerania (MV) during the summer months, and awareness campaigns for visitors [25]. In the federal state of Schleswig-Holstein (SH), samples have only been collected following reported cases of vibriosis and during irregular scientific projects [26].

Advances in remote sensing and model products provide opportunities for modelling of *Vibrio* abundances and infection risks in near real-time (NRT) [27]. One example is the *Vibrio* Map Viewer provided by the European Centre for Disease Prevention and Control (https://geoportal.ecdc.europa.eu/vibriomapviewer/, accessed on: 18 January 2023). It provides a species-independent relative *Vibrio* infection risk index. The index is calculated based on sea surface temperature (SST) and sea surface salinity (SSS) from remote sensing products, as well as ocean reanalysis and forecast products [16]. However, this species-independent approach may be inaccurate as individual *Vibrio* spp. show different responses to the same environmental conditions [17,21,28,29]. Moreover, as vibriosis infections are likely to be under-reported and infection risks have sociological, demographic, and behavioural components [12,13,17,30], modelling *Vibrio* occurrence probabilities or quantities of a certain species may provide more robust results for NRT modelling.

Despite suggestions that creating an operational NRT modelling system for *V. vulnificus* quantities in the Baltic Sea would be a step towards better management of the increasing risks of *Vibrio*-related public health issues in the future [12,13,15,17,22,27], no such attempts have been made. This study aims to pave the way towards a spatially resolved monitoring system. Such a monitoring system has to rely on observations from satellites or on model outputs. In this study, we test whether we can use a hydrodynamic model, a biogeochemical model, and a meteorological reanalysis to identify known ecological characteristics of *V. vulnificus* by coupling the model data with more than 600 samples of *V. vulnificus*. Moreover, we identify parameters particularly suited for predicting *V. vulnificus* abundances in the western Baltic Sea by applying a novel network detection method, the St. Nicolas House Analysis [31]. We discuss the suitability of the applied model data for an NRT modelling system for *V. vulnificus* and offer suggestions for improvements. Moreover, using a time series of 27 years of modelled daily SST, based on validated hindcast simulations, we attempt to detect the footprint of climate change on *V. vulnificus* season length and detect hotspots of frequent *V. vulnificus* occurrence along the German Baltic coast.

## 2. Materials and Methods

The following section outlines the study area, materials, and methodology used to identify the environmental drivers of *V. vulnificus* in the south-western Baltic Sea. A list of abbreviations and acronyms is provided at the end of the article.

### 2.1. Study Area

**Figure 1:**
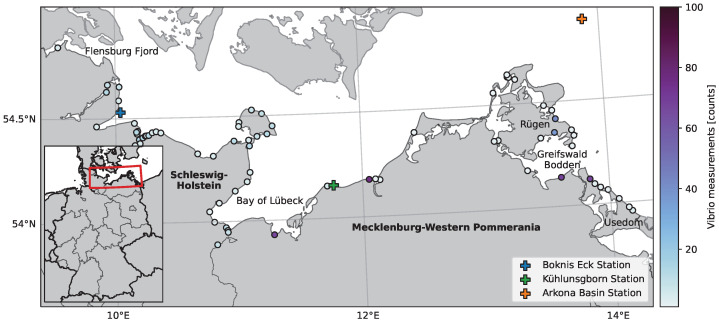
Locations of all *Vibrio vulnificus* sampling points along the German Baltic coast used in this study. The colour coding of the dots indicates the number of samples available per location between 2008 and 2019. The crosses indicate the positions of the in situ stations at which the hydrodynamic model was validated. Labelled locations are important for results and discussion.

The German coast of the Baltic Sea is characterised by large bights, the “Schlei” (a narrow inlet in the north-west of the study region of ∼30 km length), fjords, and the shallow “Bodden” in the east (Figure 1). The latter are briny, lagoon-like water bodies with complex morphologies, which naturally experience limited mixing between the sea and river estuaries. The Baltic Sea’s salinity relies solely on inflow events from North Sea water through the Kattegat. Hence, a strong salinity gradient from west to east is present in our study area. In the northwest of the study area, the average SSS is around 16–19 PSU; however, it can vary significantly due to wind-driven currents [32,33]. Towards the east of the study area, the mean SSS decreases along with its variability to values as low as 7 PSU in the open Baltic Sea. In the inlets, estuaries, and Bodden, the influx of riverine freshwater reduces the salinity even further [34].

SST shows strong seasonal dynamics. During the summer months, SST reaches averages of 17 °C in the open sea and up to 20 °C in sheltered and shallow areas. Heatwaves may cause positive SST anomalies of up to 5 °C, as observed in 2018 [12,22]. The combination of SST during the summer months and moderate salinity provides ideal conditions for *V. vulnificus* [14,20]. Moreover, large parts of the Baltic Sea are affected by eutrophication, indicating high nutrient availability [35].

### 2.2. V. vulnificus Quantification

The *Vibrio* datasets used in this study were provided by the Ministry of Social Affairs, Youth, Family, Senior Citizens, Integration and Equality Schleswig-Holstein (SoZMI), and the State Agency for Health and Social Affairs Mecklenburg–Western Pomerania (LAGuS). The former dataset consists of samples taken along the Baltic coast of the German state of Schleswig-Holstein (SH) between 2014 and 2021. The latter provides measurements from Mecklenburg–Western Pomerania (MV) between 2008 and 2021. Samples were taken during several independent monitoring projects of the federal states, which used different sampling periods and cultivation-based identification and quantification of *Vibrio* spp., as described in the following. The SH dataset contains samples from a campaign in 2014 and incident-based samples from 2015 to 2021. Incident-based samples were taken after reported cases of *Vibrio* infections. For *Vibrio* quantification and species identification, 250 mL of water was sampled from bathing areas at 30 cm below the surface according to SH bathing water directive BadGewQualV SH [36]. Moreover, 1–2 mL of the sample were filtered through membrane filters with a pore size of 0.45 µM (Millipore, Burlington, MA, USA). Filters and 200 µL of an undiluted sample were plated onto CHROMagar^TM^ Vibrio (CHROMagar, Paris, France) and incubated for 24 h at 36 °C and 5% CO${}_{2}$. Colourimetrically positive colonies were subcultured onto Marineagar (Difco^TM^ Marine Broth with 1.5% Agar–Agar, both BD Diagnostics, Franklin Lakes, NJ, USA) for a further 24 h. The colonies of *Vibrio* species (*V. parahaemolyticus*, *V. vulnificus*, and *V. cholerae*) were counted, and colony-forming units (CFUs) extrapolated for 1 ml seawater (CFU/mL). *Vibrio* identity was verified using individual colonies and a MALDI-TOF analysis (see [37] for further information on sampling, cultivation, and identification of *Vibrio*). In MV, water samples were taken every 2 weeks at 7 stations starting in 2008. Sampling began each summer after a threshold water temperature of 17–18 °C was reached [25]. *Vibrio* identification and quantification in water samples was done using a cultivation-based approach, similar to the SH dataset. For a description of the cultivation, refer to [38]. While the CFU/mL was calculated in the SH dataset, the MV dataset estimated *V. vulnificus* abundance in powers of 10 (the most probable number technique). This entailed screening *Vibrio* growth along a dilution series and recording the highest dilution with visible colonies. The experiments were conducted in triplicate. To make the SH and MV datasets comparable, the SH records were converted into corresponding powers of ten.

Quantitative measurements were mostly only available for *V. vulnificus*, which is also the dominant potentially human-pathogenic *Vibrio* spp. in the south-western Baltic Sea [22]. Other *Vibrio* spp. were only recorded as present/absent in a sample. Thus, we focused on working with samples of *V. vulnificus* only.

### 2.3. Environmental Data

To explore the relationship between environmental factors and *V. vulnificus* response, data for 11 parameters were extracted from models and reanalysis, including freely available biogeochemical and meteorological reanalysis products (Table 1). Special emphasis was put on SST and SSS, which have been identified as the most important parameters driving *V. vulnificus* abundance in several studies (e.g., reference [2] and references therein). To reflect this, a high-resolution hydrodynamic model was used to resolve coastal areas and narrow inlets. The following sections will briefly present the different model systems.

#### 2.3.1. High-Resolution Hydrodynamic Model

The General Estuarine Transport Model (GETM) [41,42] was used to simulate SST and SSS in the western Baltic Sea from 1995 to 2021. The 3D simulations used a horizontal resolution of 200 m on a spherical grid with 35 depth layers, which were adapted to vertical density gradients [42,43]. Turbulence closure (second-order, k-ε) was implemented by using the General Ocean Turbulence Model [44,45]. Boundary conditions at the open boundaries were taken from a larger-scale model of the Baltic Sea (see [32] for details).

Model results were validated with in situ data from three in situ sampling stations (Figure 1). At Boknis Eck, the modelled data were compared to monthly measurements of temperature and salinity at 1 m depth (https://www.bokniseck.de/, accessed on: 27 November 2022) [46]. The Leibniz Institute for Baltic Sea Research Rostock, Germany, provided daily mean water temperatures for the Kühlungsborn station measured at a 1 m depth. At the Arkona Basin station, part of the Marine Environmental Monitoring Network [47], daily averages of the shallowest available measurement depths of water temperature and salinity (0.5 m and 2 m, respectively) were used in the validation exercise. To asses the model accuracy, we calculated the Pearson correlation coefficient (*r*), mean absolute error (MAE), and bias between modelled and observed data.

#### 2.3.2. Biogeochemical Data

Data on nutrients, dissolved oxygen (O${}_{2}$), and Chl (a proxy for phytoplankton biomass [48]) were taken from a biogeochemical reanalysis product for the Baltic Sea produced by the Copernicus Marine Environment Monitoring Service CMEMS [39]. The reanalysis product is based on the Nucleus for European Modelling of the Ocean (NEMO) circulation model in the NEMO-Nordic configuration [49,50], coupled with the Swedish Coastal and Ocean Biogeochemical model [51,52]. The model is forced with meteorological data and observations, including SST charts and in situ measurements of temperatures and several chemical parameters [53]. The model domain covers the entire Baltic Sea and its transition to the North Sea. It resolves up to 56 depth layers and has provided daily averages of NO${}_{3}$, PO${}_{4}$, NH${}_{4}$, O${}_{2}$, and Chl since 1993 at a spatial resolution of approximately 4 × 4 km.

We accessed the dataset through CMEMS OPeNDAP Python API and downloaded all daily averages of the surface layer in our study region since the beginning of the *V. vulnificus* sampling program in 2008.

#### 2.3.3. Meteorological Data

The COSMO-REA6 dataset is a reanalysis product from the German Weather Service DWD [40]. It is based on the COSMO (Consortium for Small-scale Modelling) numerical weather prediction model and a data assimilation scheme that incorporates observations from various sources, such as weather stations, ships, and aircraft. The model domain covers Europe and northern Africa with a spatial resolution of 6 × 6 km. Hourly data are available for the period between 1995 and August 2019.

We downloaded daily averages of surface air temperature (SAT), total precipitation, solar irradiation, and wind speed (calculated from u and v components) for the study period (2008–2019) from the open data server of the DWD (https://opendata.dwd.de/climate_environment/REA/, accessed on: 6 December 2022).

#### 2.3.4. Aggregation of Environmental Data and Identification of Lead Time

Several studies indicate a lead time between some environmental parameters (e.g., air temperature) and *V. vulnificus* abundance [21,38,54]. For this reason, time series of all environmental parameters from the 30 days before a *V. vulnificus* sampling time were extracted for each sampling to calculate the lead time with the highest predictive power for each parameter. Due to the spatial resolution of the GETM and CMEMS Biogeochemistry Reanalysis products (200 × 200 m and 4 × 4 km, respectively), valid data was not always available directly at the coastal sampling locations. In such situations, all data within a predefined radius around the sampling location were averaged. The search distance was increased iteratively until valid predictions were found or a maximum radius of 1.5 × the grid cell resolution was reached.

**Figure 2:**
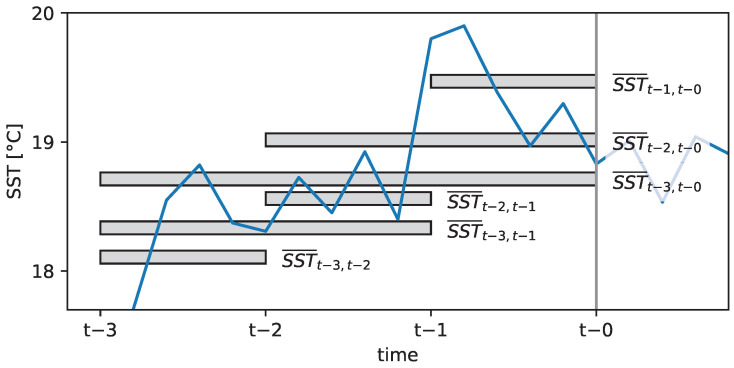
Illustration of the moving window of a flexible size for a time series of sea surface temperature (SST). *V. vulnificus* sampling took place at time *t* − 0. The grey bars on the time axis indicate the subset of the time series covered by the different windows, while the location on the SST axis indicates the average SST within the different periods. ${\bar{\mathrm{SST}}}_{t-3,t-1}$ denotes the average SST in the time window between *t*− 3 and *t*− 1.

The 30-day time series were analysed using a moving window of flexible size (Figure 2). For each window, the average (for precipitation, the sum) was calculated, correlated with *V. vulnificus* quantities, and plotted in correlation matrices. Spearman’s rank correlation was used to account for potential non-linearity of underlying processes. The window with the highest correlation coefficient was generally used as the time lag in further analysis.

It has already been observed that *V. vulnificus* quantities not only depend on absolute values of a parameter, but also on the duration of an event [19,55]. Therefore, we chose to not only consider absolute values of environmental parameters but also their trends by estimating the slope of a linear least squares fit. The trend was calculated for each moving window of flexible size, and we identified the window with the highest correlation coefficient using Spearman’s rank correlation.

Previous studies in temperate regions have shown that *V. vulnificus* enters a viable but non-culturable state (VBNC), which is a survival strategy altering their metabolism if water temperatures drop below ∼13–15 °C [56]. For resuscitation, the cells require considerably higher temperatures (∼17–20 °C). To capture this seasonal dynamic, an additional parameter containing the maximum SST of the past weeks and months was added. The best window was determined with the same workflow as described above, but on a time series covering the period of 30 to 300 days before sampling.

In the following, lag windows of variables will be denoted as, for instance, SST_*mean*12–15_. This stands for the average SST between 12 and 15 days before Vibrio sampling.

### 2.4. Statistical Analysis

Initially, we tested the ability of our models to reproduce known ecological characteristics of *V. vulnificus* by calculating Spearman’s rank correlation coefficient ($r_{s}$) between all parameters and plotting *V. vulnificus* quantities over SST and SSS in a bubble plot. Ecological preferences and distribution boundaries were then compared with literature studies from temperate regions. We then identified parameters associated with *V. vulnificus* quantities using the Saint Nicolas House Analysis (SNHA). SNHA provides an approach for initial data analysis by visualizing associations between variables in non-linear multivariate data [31]. It ranks absolute bivariate correlation coefficients in descending order according to the magnitude and creates hierarchical association chains. These association chains must also be reversible, i.e., the same order must be derived if the end node (i.e., an environmental parameter in our study) of a chain becomes the start node. Superimposing all detected chains creates a network graph, which can be used to characterize and visualize dependencies between interacting variables, as described by Hermanussen et al. [57]. In contrast to similar approaches, SNHA is non-parametric, robust to outliers, and relatively robust against spurious significance [31,57].

Recently, Hake et al. [58] proposed an improvement for the detection of branched association chains in densely interconnected networks using a bootstrapping routine. Bootstrapping involves randomly selecting samples with replacements from the original dataset. In each re-sampling, SNHA is applied, and the detected edges (i.e., connections between nodes) are counted across all re-samplings. An edge becomes significant if it is detected more than λ times. λ is estimated using a binomial test and depends mainly on the number of bootstrap iterations and the probability of success (i.e., the probability an edge is falsely detected).

SNHA was applied to the complete dataset of *V. vulnificus* samples and environmental parameters to unveil a network between the different parameters. We used the Python implementation SNHA4py (https://github.com/thake93/snha4py, accessed on: 3 January 2023, v20230103) [58] and the bootstrapping routine with 100 iterations. The binomial test with a probability of success of 0.035 (derived empirically by Hake et al. [58]) suggested that edge predictions made in more than 8% of all iterations are statistically significant (p<0.05).

Preliminary results identified SST as the most important parameter determining *V. vulnificus* quantity. Our high-resolution SST product provides the unprecedented opportunity to identify hotspots with frequently favourable SST and regions with high warming rates since 1995. To identify these regions, we first calculated the *V. vulnificus* occurrence probability with logistic regression on our *V. vulnificus* samples and corresponding modelled SST values from our GETM realisation. Next, we counted the days in each year where SST exceeded the temperature of the 33% probability of *V. vulnificus* occurrence (from hereon referred to as season length; the selection of the threshold will be discussed later). Trends were estimated with Sen’s slope, and their statistical significance was ensured with the Mann–Kendall test using the pyMannKendall package [59].

## 3. Results

### 3.1. Validation of the Hydrodynamic Model

**Figure 3:**
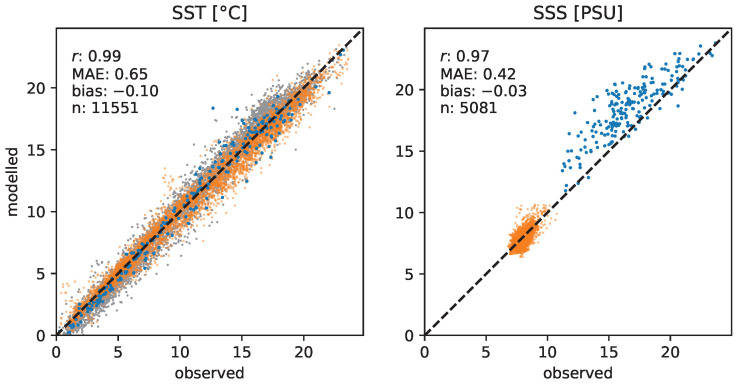
Validation of SST and SSS products of the GETM realization with observations at stations Boknis Eck (blue), Kühlunsborn (gray), and Arkona Basin (orange). Note that at Kühlungsborn, only SST is measured. *r*: Pearson correlation coefficient. MAE: mean absolute error. n: number of samples.

Validation of the high-resolution hydrodynamic model demonstrates the accuracy of both SST and SSS products (Figure 3). Both parameters are strongly correlated with in situ data (*r* of 0.99 and 0.97 for SST and SSS, respectively). However, the model appears to slightly underestimate SST (bias = −0.1 °C, MAE = 0.66 °C). For SSS, the MAE is approximately 0.4 PSU and the bias is negligible (−0.03 PSU), although SSS at Boknis Eck appears to be moderately overestimated (bias of 1.88 PSU).

**Figure 4:**
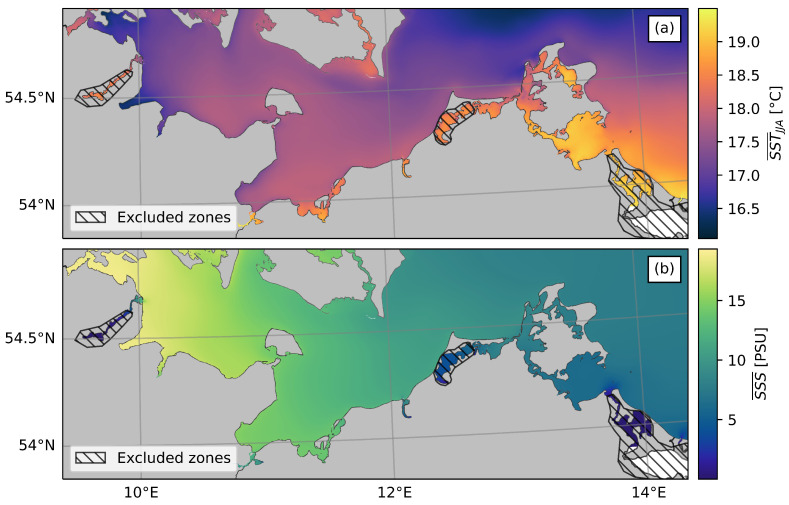
Results of the GETM realisation. (**a**) Average SST in summer months (JJA) in the study period. (**b**) Average SSS in the study period. Dashed areas show regions with unreliable SSS results, which were excluded in the following analysis.

Figure 4 shows the average SST during the summer months (June–August) and the average SSS. The SST patterns appear reasonable, with higher temperatures in shallow and sheltered bays and lower temperatures in offshore areas with greater depths. In the northwest of the study area, relatively low SSTs close to the shore can be attributed to frequent local upwelling events during the summer months [60]. The SSS climatology closely depicts the salinity gradient along the German Baltic coast. However, the model results indicate low salinity (nearly 0 PSU) for three narrow inlets (areas marked with hashed lines in Figure 4), which contradicts published salinity measurements from these areas [34]. This discrepancy may be caused by the model’s underestimation of the water exchange between the open Baltic Sea and these bays, as the narrowest inlet of all three areas is smaller than the model’s grid resolution of 200 m. Therefore, these specific bays were excluded from the subsequent analysis.

### 3.2. Analysis of Lag Windows and SNHA

Data of all environmental parameters were available for a total of 621 *V. vulnificus* samples (408 in MV and 213 in SH). For each environmental parameter, we created correlation matrices with *V. vulnificus* quantities. In most cases, the lag window with the highest $r_{s}$ was chosen for further analysis. For the parameters ${\mathrm{SST}}_{trend}$, ${\mathrm{PO}}_{4trend}$, and ${\mathrm{Chl}}_{mean}$, we selected considerably longer window lengths with slightly lower $r_{s}$ (see Table A1 for details).

With the selected lag windows, we calculated a correlation matrix with *V. vulnificus* quantities and all environmental parameters (Figure A2). All environmental parameters, except Chl_*mean*6–15_ (i.e., mean Chl between 6 and 15 days before *Vibrio* sampling), correlate significantly with *V. vulnificus* quantity (*p* > 0.05), albeit most $r_{s}$ are low. Parameters with the highest absolute $r_{s}$ are SST_*mean*0–11_, SAT_*mean*0–16_, O_2*mean*0–0_ and SSS_*mean*0–6_ ($r_{s}$ of 0.55, 0.49, −0.37, and −0.35, respectively).

**Figure 5:**
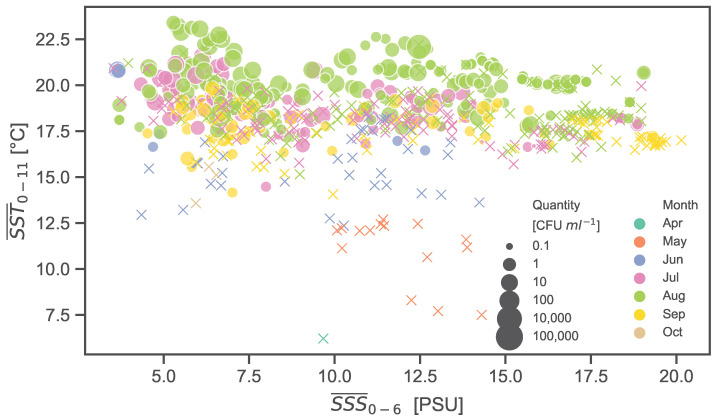
Bubble plot showing *V. vulnificus* quantities (indicated by bubble size) in relation to average SST and SSS of the days before the sampling event. Negative *V. vulnificus* samples are indicated with crosses. The sampling month is denoted by the colour of the crosses and bubbles.

Figure 5 shows the abundance of *V. vulnificus* in relation to both SST and SSS. The probability that *V. vulnificus* occurs increases with higher SST, starting from ∼17 °C. At 19 °C, *V. vulnificus* was detected in about 60% of all samples. The lowest temperature at which *V. vulnificus* could be detected was 14.1 °C. In contrast to temperature, *V. vulnificus* was observed across almost the entire salinity gradient of the study area (3.7–19.4 PSU).

**Figure 6:**
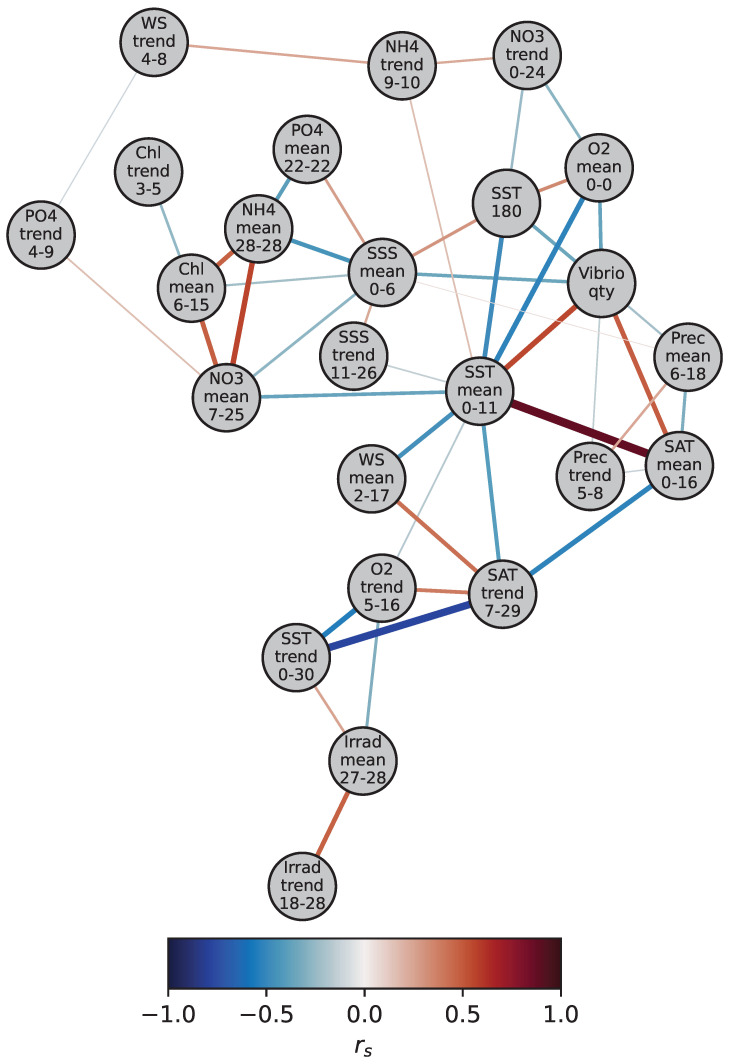
The network between the *V. vulnificus* quantity and environmental parameters derived with the Saint Nicolas House Analysis (SNHA). Each circle represents a variable (see Table 1 for abbreviations). The last row in the circles represents the time window in days. The edge colour represents Spearman’s rank correlation coefficient between parameters ($r_{s}$).

We applied SNHA to derive a network between all parameters (Figure 6). The mean sea surface temperature (more specifically SST_*mean*0–11_) forms the centre of the network and is connected to 10 other parameters, including biogeochemical, meteorological, and physical parameters, as well as the *V. vulnificus* quantity. The biogeochemical parameters sharing an edge with SST are mean oxygen (O${}_{2}$_*mean*0–0_), mean nitrate (NO${}_{3}$_*mean*7–25_), mean ammonium (NH${}_{4}$_*trend*9–10_), and the oxygen trend (O${}_{2}$_*trend*5–16_). The meteorological parameters include mean surface air temperature (SAT_*mean*0–16_), wind speed (WS_*mean*2–17_), and the trend of surface air temperature (SAT_*trend*7–29_). The physical parameters connected to the mean sea surface temperature are the sea surface temperature 180 days before sampling (SST${}_{180}$) and the trend of sea surface salinity (SSS_*trend*11–26_). With an $r_{s}$ of 0.88, the average sea surface temperature (SST_*mean*0–11_) and surface air temperature (SAT_*mean*0–16_) are closely related.

Direct edges to *V. vulnificus* were inferred from seven parameters. Among them are mean sea surface temperature (SST_*mean*0–11_, $r_{s}$ of 0.55), mean surface air temperature (SAT_*mean*0–16_, $r_{s}$ of 0.49), mean oxygen concentration (O${}_{2}$_*mean*0–0_, $r_{s}$ of −0.37), mean sea surface salinity (SSS_*mean*0–6_, $r_{s}$ of −0.35), surface temperature 180 days before sampling (SST${}_{180}$, $r_{s}$ of −0.35), average precipitation (Prec_*mean*6–18_, $r_{s}$ of −0.21), and the trend of precipitation (Prec_*trend*5–8_, $r_{s}$ of −0.16). The correlations of all these edges are significant at the *p* < 0.001 level. There is no direct edge between *V. vulnificus* quantity and nutrients or Chl. However, mean ammonium (NH_4*mean*28–28_), phosphate (PO_4*mean*22–22_), nitrate (NO_3*mean*7–25_), and chlorophyll (Chl_*mean*6–15_) have a link to mean sea surface salinity (SSS_*mean*0–6_), while mean nitrate (NO_3*mean*7–25_) and the trend of ammonium (NH_4*trend*9–10_) are connected to the average sea surface temperature SST_*mean*0–11_. These nutrients are, thus, indirectly connected to the *V. vulnificus* quantity.

### 3.3. Trends of the V. vulnificus Season Length

**Figure A1:**
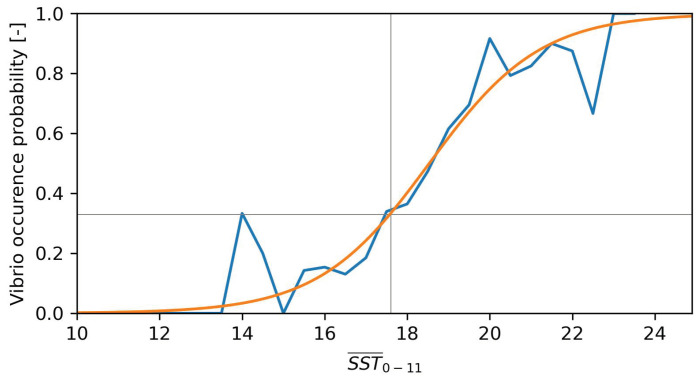
Probability of the *V. vulnificus* occurrence over ${\mathrm{SST}}_{mean0–11}$. The blue line shows the probability of occurrence of all samples calculated per 0.5 °C and the orange line is the logistic regression.

To describe the length of the *V. vulnificus* season, we calculated the occurrence probability based on SST with logistic regression. The fit reveals a slow increase of occurrence probability up to ∼17 °C. At 17.6 °C, the probability reaches 33% and increases sharply by about 20% per degree (Figure A1).

**Figure 7:**
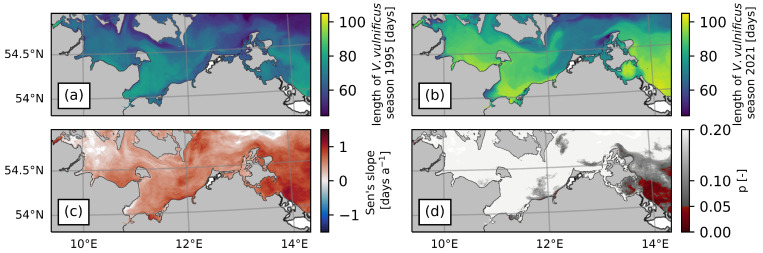
*V. vulnificus* season length and trends calculated based on SST between 1995 and 2021. (**a**) Season length in 1995. (**b**) Season length in 2021. (**c**) Trend estimated with the Theil–Sen estimator. (**d**) Significance (p) of the trend calculated with the Mann–Kendall test.

Figure 7 displays the *V. vulnificus* season length (i.e., days with SST > 17.6 °C) for 1995 (a) and 2021 (b). Apart from the trend towards a longer season in 2021, the season length exhibits similar geographic patterns in both years. Longer than average seasons occurred, for example, in the inner Flensburg Fjord in the north-west of the study region, the Bay of Lübeck in the centre, and the easternmost areas of the study region (see labels in Figure 1 for the locations of these regions). Relatively short seasons can be observed in some inlets in the west of the study region, near the Danish islands of Lolland and Falster, and in the north-west of the island Rügen. Figure 7c indicates a trend towards a longer *V. vulnificus* season in virtually the entire German Baltic Sea, although in most areas, the trends are not significant (Figure 7d). The most distinct area with a significant increase in season length is located in the east of the study area, close to the island of Usedom. Smaller areas with significant increases are located in sheltered and shallow bays with limited water exchange (e.g., Greifswald Bodden or Flensburg Fjord) and in the extension of the estuary of the river Warnow. The fastest season expansion was calculated for the inner Flensburg Fjord, increasing at a rate of up to 1.4 days per year.

## 4. Discussion

This study represents an initial step towards developing a spatially explicit near real-time (NRT) modelling system for the German Baltic Sea by combining more than 600 *V. vulnificus* samples from the Baltic coast with modelled data of environmental parameters such as SST, SSS, and nutrient concentrations. On the one hand, this allows evaluating the suitability of the models to resolve key ecological characteristics of *V. vulnificus*. On the other, it can pinpoint ecological parameters that are best suited for predicting *V. vulnificus* occurrences in the Baltic Sea using a statistical model.

### 4.1. Ecological Characteristics of V. vulnificus in the South-Western Baltic Sea

Our results largely confirm the important constraining influence of SST on *V. vulnificus* abundance in regions with a temperature-driven seasonal cycle, e.g., [20,61,62]. In fact, we identified SST${}_{mean0-11}$ to be the most limiting parameter in our region of study. The probability of *V. vulnificus* occurrence increased rapidly once SST exceeded ∼17 °C. The highest numbers of *V. vulnificus* CFUs were observed when SST was above 20 °C. These dynamics agree well with reports from other studies, e.g., [21,22,63].

After proliferating in the summer months, *V. vulnificus* can persist at lower temperatures before entering the VBNC state. Oliver et al. [56] reported that the transition into the VBNC state occurs at around 15 °C SST. These results have been confirmed by studies in Barnegat Bay, USA (12 °C in [20]) and in the German North Sea (14 °C in [21] and 13 °C in [29]). The 14 °C detection limit determined by the model in this study is thereby confirmed by commonly observed experimental values. To capture the seasonal effect of a larger tolerance of *V. vulnificus* towards lower water temperatures in autumn before entering the VBNC state, we included SST 180 days before sample acquisition (SST${}_{180}$) as a parameter in the SNHA analysis. Indeed, SNHA inferred an edge between SST${}_{180}$ and *V. vulnificus* quantity. This indicates that SST${}_{180}$ takes into consideration different aspects of the variability in *V. vulnificus* quantities, such as season, which are not detected by SST with no or minimal lag. It may, therefore, be a valuable additional predictor in a statistical NRT model.

SNHA also detected an edge between *V. vulnificus* quantity and SAT_*mean*0–16_. Given the strong correlation between SAT_*mean*0–16_ and SST_*mean*0–11_ ($r_{s}$ = 0.88), it is likely that the edge between *V. vulnificus* quantity and SAT_*mean*0–16_ represents no direct causal relationship. Instead, it may derive from an indirect mechanism through the coupling of SAT and SST, which is particularly strong in the Baltic Sea [64]. However, since SNHA inferred edges between *V. vulnificus* quantity and both SAT_*mean*0–16_ and SST_*mean*0–11_, these two parameters may capture different aspects of environmental and *V. vulnificus* variability. Therefore, they may be further useful in statistical modelling exercises [54]. The same holds true for the edge between *V. vulnificus* quantity and O_2*mean*0–0_, which has been observed elsewhere [65,66,67]. While changes in oxygen saturation can affect the metabolism of *V. vulnificus* [68], this edge more likely reflects the strong effects of temperature on oxygen solubility.

In addition to the strong relation of *V. vulnificus* abundance with SST, many studies have highlighted SSS outside a range of approximately 4–25 PSU as a limiting factor [20,21,22,66,67]. Considering the SSS gradient in the German Baltic Sea, which ranges from ∼20 PSU in the north-west to ∼3.5 PSU in the eastern lagoons of the studied region, it is likely that SSS does not limit the occurrence of *V. vulnificus*. However, optimal growth conditions were reported at around 10 PSU [20,23,63], which indicates that the lower SSS in the eastern part of the study region allows higher growth rates. This pattern is also evident from our *V. vulnificus* data, where 63 out of 69 samples with >100 CFU/mL were acquired in MV.

Weak negative correlations between *V. vulnificus* quantity and precipitation ($r_{s}$ of −0.21 and −0.16 for Prec_*mean*6–18_ and Prec_*trend*5–8_) differ from findings in other regions, such as e.g., the North Sea [21], Mediterranean Sea [23], or Hawaii [54]. In all three examples, precipitation or increased river discharge diluted coastal waters with a SSS of >30 PSU to brackish water, thereby creating favourable conditions for *V. vulnificus*. Hence, precipitation appears to be more important in regions where high SSS limits *V. vulnificus* growth. In the brackish Baltic Sea, precipitation may simply reflect variations of the weather in the days and weeks before the sampling. Further, the GETM hydrodynamic model takes the effect of precipitation on SSS into account. This means that potential effects of precipitation on salinity are already included in the SSS parameter.

**Figure A2:**
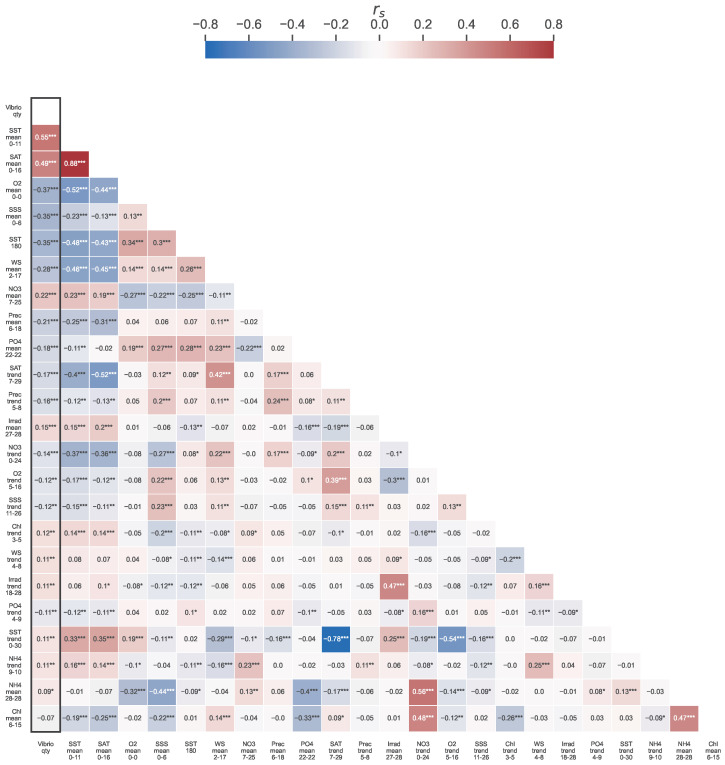
Correlation matrix ($r_{s}$) between *V. vulnificus* abundance and all environmental parameters. Asterisks after $r_{s}$ indicate the significance level (*: p≤0.05, **: p≤0.01, ***: p≤0.001).

Although significant correlations between *V. vulnificus* and different nutrient concentrations exist (Figure A2), SNHA detects no direct edge between them. Instead, nutrient parameters and *V. vulnificus* quantity connect only through SST_*mean*0–11_ and SSS_*mean*0–6_. In contrast, Bullington et al. [54] noted that nutrient availability plays a crucial role in limiting *V. vulnificus* growth around O’ahu, Hawaii, USA. However, this can be explained by the small annual SST variability in tropical regions, which allows other parameters to emerge as regulating factors [8]. In the Baltic Sea, nutrients show distinct annual cycles that correlate with SST [69]. As SST is such a strong driver of the environmental processes in the Baltic Sea, its impact may outweigh any visible influence of nutrient availability on *V. vulnificus* abundance in the model. Furthermore, it cannot be ruled out that the nutrient model product, with its relatively coarse spatial resolution of 4 × 4 km, limits our analysis. An in-depth investigation that includes in situ nutrient samples alongside *Vibrio* quantification for validation of the model may help to unveil the influence of nutrient availability on *V. vulnificus* in the Baltic Sea.

Resuspension could be an important factor regarding seeding and dispersal of *Vibrio* from the sediment back into the water column [70]. To estimate sediment resuspension in the study, the wind speed parameter was considered. However, the seasonality of wind events seems to outweigh the potential effect on *V. vulnificus* quantities in the region. Moreover, resuspension depends on several factors such as fetch, water depth, and grain size. Recently, DeLuca et al. [71] improved their ability to predict *V. parahaemolyticus* abundance by using remote sensing data on total suspended solids as a proxy for resuspension instead of wind speed. Therefore, it may be worthwhile to test such data in future iterations of the *V. vulnificus* model.

### 4.2. Perspectives for NRT Monitoring

As discussed, the edges identified by our time lag analysis and subsequent SNHA do not necessarily represent causal effects. However, they imply that a parameter with a certain lag window would provide additional information to a statistical model. We, therefore, conclude that this workflow can help to select important parameters that may be used as features in a future model. When constructing and training a model, feature importance measures such as LIME or SHAP may help to refine feature and lag window selection further.

Our results highlight the importance of SST and, to a lesser extent, SSS on *V. vulnificus*. Given the high accuracy of the hydrodynamic model in open waters, it appears well-suited for NRT modelling. However, the inconsistencies in narrow inlets need further investigation, particularly as these areas are important tourist destinations. Improving the parameterisation for freshwater inflow and using configurations with even higher resolution models may help to resolve the areas that had to be excluded from this study. The trade-off between product resolution and computational cost must be carefully considered, as a resolution of 200 m is not yet regularly run for NRT due to its high computational demand. One possible solution may be a nested model structure with a coarser grid resolution in open water and a higher resolution in inlets, as shown in [32]. However, remote sensing data and products may be alternatives for future *V. vulnificus* NRT monitoring as they enable the detection and mapping of suitable environmental conditions, such as SST and SSS, and provide cost-effective and efficient measures of large areas over time.

Reanalysis products were used for meteorological and biogeochemical parameters. Both reanalyses were chosen based on their temporal coverage, which overlaps with most of the *V. vulnificus* time series. However, it is important to note that these products cannot be readily incorporated into an NRT modelling system. The biogeochemical reanalysis is accessible only after a delay of one year from real-time, while the meteorological reanalysis was only available until August 2019. However, alternatives exist, such as a new biogeochemical forecast from CMEMS [72], the openly available weather model ICON-D2 [73], and the MET Nordic Analysis, a meteorological reanalysis of the Norwegian Meteorological Institute (https://github.com/metno/NWPdocs/wiki/MET-Nordic-dataset, accessed on: 29 October 2022). These alternatives have only become available in recent years and do not cover the entire period of *Vibrio* monitoring. Therefore, thorough model comparisons would be required if model training and prediction are carried out on different datasets.

It is crucial to keep in mind that the quality of the underlying data limits the effectiveness of any statistical model. In this study, we were able to generate a dataset with more than 600 samples, although these samples were acquired in two different federal states with slightly different sampling protocols. While both states assessed *V. vulnificus* quantities through cultivation and CFU count/ml, differences in experimental approaches between laboratories exist. The datasets were homogenised, but this came at the expense of losing the continuous scale of the data from SH in favour of the less detailed categorical system from MV. Moreover, existing sampling strategies primarily monitor *Vibrio* quantities during conditions favourable for *V. vulnificus* or after reported infections. Consequently, July and August contribute to over 70% of all the samples. The accuracy of a future iteration of the NRT model would certainly benefit from additional samples derived from consistent, year-round sampling campaigns. Such sampling efforts would also provide the opportunity to extend our approach to other (potentially human) pathogenic *Vibrio* species, such as *V. parahaemolyticus*, *V. cholerae* or *V. fluvialis*, which have been studied less intensely in the German Baltic Sea [22,74].

In another attempt to gain additional *Vibrio* data to train the NRT models, an additional resource may be found in the DNA databases, such as the NCBI Sequence Read Archive (https://www.ncbi.nlm.nih.gov/sra, accessed on: 20 February 2023). These databases contain large amounts of marker-gene or metagenomic sequencing data from around the world, and could be compiled to search for relative abundances (proportions) of *Vibrio* amongst microbial communities. At the very least, the data could provide presence/absence-based information for different sampling locations around the globe. This approach would, however, require considerable computational efforts, as well as close curating of the datasets, which are beyond the scope of this study.

### 4.3. Climate Change

Globally, the Baltic Sea is the marine region with the highest rate of warming [24]. Along the German coast, the highest increase in SST is projected for spring and fall [75]. Observations at Boknis Eck Time Series Station support this trend and further note an increase in the frequency of heat anomalies in the summer [46]. The effects of climate change on the abundance of *V. vulnificus* and their distribution have been widely discussed and can already be observed. For instance, higher numbers of vibriosis cases are reported during exceptionally warm summers [12,13]. The present study suggests that the season when *V. vulnificus* is likely to occur has been extended almost throughout the entire southwestern Baltic Sea since 1995, due to the rising water temperatures. In some areas, the season has extended at rates exceeding one day per year. These trends, however, are significant only in areas that experience the highest rates of warming. These mainly include sheltered and shallow bays, as well as the area east of the island of Usedom. East of Usedom, the water is relatively turbid, leading to more intense heating of the water [76,77]. In shallow bays, such as the Greifswald Bodden, bathymetry limits the water exchange [34], thereby causing higher warming rates. The Greifswald Bodden is also the area for which Brennholt et al. [38] projected the strongest increase in the *V. vulnificus* season length, defined as days with SST exceeding 17 °C. In other regions, trends in season length remain insignificant, likely due to the underlying climate variability obscuring them.

The influence of climate variability also explains our rather arbitrary choice for the SST threshold at a *V. vulnificus* occurrence probability of 33% (i.e., SST > 17.6 °C). When we used higher probabilities as the threshold (e.g., 50%, i.e., SST > 18.5 °C), trends remained insignificant in almost the entire study area. We suspect that this was due to an increase in variance as the temperature threshold rose, likely due to an increased impact of extreme events. Under such conditions, the Mann–Kendall test may be unable to identify comparably small slopes [78]. While the estimated *V. vulnificus* season length requires further improvement, the influence of climate change on the occurrence of *V. vulnificus* was evident from our data. As the season is closely linked to water temperature and, therefore, the public bathing season, an increase in human exposure to *V. vulnificus* can be expected [17].

Increased SST and a longer *V. vulnificus* season may also lead to increased stress on densely colonised aqua- and larvicultures by members of the *V. vulnificus*, which primarily infect marine hosts. While this can cause mass mortalities within the aquacultures themselves, derived seafood can provide an additional source for human contact and subsequent vibriosis [79,80]. Infecting humans as secondary hosts is not only limited to *V. vulnificus*, but also includes other zoonotic *Vibrio* members, such as *V. parahaemolyticus* [81] or (rarely) *V. alginolyticus* [82]. It may, therefore, be advantageous to increase the scope of Vibrio monitoring to include a variety of *Vibrio* species and/or serotypes.

## 5. Conclusions

By analysing environmental data from models and reanalyses, we found key ecological factors for predicting *V. vulnificus* quantities in the German Baltic Sea. The most significant factors were SST, SAT, SSS, dissolved oxygen, and precipitation. This is the first step in creating an NRT model to forecast the occurrence and quantities of *V. vulnificus*. Given that climate change is increasing human exposure to *V. vulnificus*, such an NRT prediction system is crucial for managing future infection risks. To develop a reliable NRT model, we advise standardising *Vibrio* spp. sampling strategies and measurement methods across all federal agencies.

## Figures and Tables

**Table 1:** Utilised datasets, environmental parameters, and their abbreviations.

Dataset	Parameter	Abbreviation	Spatial Resolution	Temporal Coverage
GETM-IOW	Sea Surface Temperature	SST	0.2 × 0.2 km	1995–2021
	Sea Surface Salinity	SSS		
CMEMS Baltic Sea Biogeochemistry Reanalysis [39]	Ammonium	NH${}_{4}$	4 × 4 km	1993–2021
	Nitrate	NO${}_{3}$		
	Phosphate	PO${}_{4}$		
	Oxygen	O${}_{2}$		
	Chlorophyll a	Chl		
COSMO-REA6 [40]	Surface Air Temperature	SAT	6 × 6 km	1995–August 2019
	Wind speed	WS		
	Precipitation	Prec		
	Solar Irradiation	Irrad		

## Data Availability

*Vibrio* data were obtained from the State Agency for Health and Social Affairs Mecklenburg–Western Pomerania (https://www.lagus.mv-regierung.de/) and the University Medical Center Schleswig Holstein (https://www.uksh.de/hygiene-kiel/) and are available upon request. Model results of the hydrodynamic model GETM are available at https://thredds-iow.io-warnemuende.de/thredds/catalogs/regions/baltic/regions/catalog_WB200m.SST.html. The Python code of the analysis and all plots are available on GitHub (https://github.com/eikeschuett/Vibrio_Baltic_Sea).

## Abbreviations

CFU	colony forming units
Chl	Chlorophyll a
CMEMS	Copernicus Marine Environment Monitoring Service
COSMO	Consortium for Small-scale Modelling
DOC	Dissolved Organic Carbon
DWD	German Weather Service
GETM	General Estuarine Transport Model
Irrad	Solar Irradiance
LAGuS	State Agency for Health and Social Affairs Mecklenburg-Western Pomerania
MAE	Mean Absolute Error
MV	Mecklenburg-Western Pommerania
NEMO	Nucleus for European Modelling of the Ocean
NH_4_	Ammonium
NO_3_	Nitrate
NRT	Near real-time
O_2_	Oxygen
PO_4_	Phosphate
Prec	Precipitation
PSU	Pratcital Salinity Unit
*r*	Pearson correlation coefficient
$r_{s}$	Spearman’s rank correlation coefficient
SAT	Surface Air Temperature
SH	Schleswig-Holstein
SNHA	Saint Nicolas House Analysis
SoZMI	Ministry of Social Affairs Youth, Family, Senior Citizens, Integration and Equality
	Schleswig-Holstein
SSS	sea surface salinity
SST	sea surface temperature
*V.*	*Vibrio*
VBNC	Viable but non-culturable state
WS	Windspeed

## Appendix A

**Table A1:** Selected lag window for each parameter and aggregation method. $r_{s}$ denotes the Spearman’s rank correlation coefficient between the environmental parameter and *V. vulnificus* quantity. In most cases, the lag window with the highest $r_{s}$ was selected. These cases are indicated as “yes” in the column “max $r_{s}$”. For all other cases, the column “max $r_{s}$” indicates the time window with the highest $r_{s}$ using the format “parameter_*start*–*end*_ ($r_{s}$)”.

Parameter	Aggregation	Lag Window		$r_{s}$	Max $r_{s}$
		Start	End		
SST	mean	0	11	0.55	yes
	trend	0	30	0.11	SST_*trend*24–25_ (0.14)
	max	180	180	−0.35	yes
SSS	mean	0	6	−0.35	yes
	trend	11	26	−0.12	yes
NH${}_{4}$	mean	28	28	0.09	yes
	trend	9	10	0.11	yes
NO${}_{3}$	mean	7	25	−0.14	yes
	trend	0	24	0.22	yes
PO${}_{4}$	mean	22	22	−0.18	yes
	trend	4	9	−0.11	PO_4*trend*20–21_ (−0.13)
O${}_{2}$	mean	0	0	−0.37	yes
	trend	5	16	−0.12	yes
Chl	mean	6	15	−0.07	Chl_*mean*15–15_ (−0.09)
	trend	3	5	−0.12	yes
SAT	mean	0	16	0.49	yes
	trend	7	29	−0.17	yes
WS	mean	2	17	−0.28	yes
	trend	4	8	0.11	yes
Prec	mean	6	18	−0.21	yes
	trend	5	8	−0.16	yes
Irrad	mean	27	28	0.15	yes
	trend	18	28	0.11	yes
